# Factors behind the prevalence of carbapenem-resistant *Klebsiella pneumoniae* in pediatric wards

**DOI:** 10.1097/MD.0000000000027186

**Published:** 2021-09-10

**Authors:** Yuxin Yang, Jia Liu, Murad Muhammad, Hanting Liu, Zongsu Min, Jing Lu, Lei Zhang, Zhonglin Chai

**Affiliations:** a Department of Pathology, Zunyi Maternity and Child Health Care Hospital, Zunyi, Guizhou, China; b Department of Diabetes, Central Clinical School, Monash University, Melbourne, Victoria, Australia; c Department of Pharmacy, Zunyi Maternity and Child Health Care Hospital, Zunyi, Guizhou, China; d Department of Surgery (RMH), University of Melbourne, Melbourne, Victoria, Australia; e China-Australia Joint Research Center for Infectious Diseases, School of Public Health, Xi’an Jiaotong University Health Science Center, Xi’an, Shanxi, China; f Department of Breast Surgery, Guizhou Provincial People's Hospital, Guiyang, Guizhou, China; g Artificial Intelligence and Modelling in Epidemiology Program, Melbourne Sexual Health Centre, Alfred Health, Melbourne, Australia; h Central Clinical School, Faculty of Medicine, Monash University, Melbourne, Australia; i Department of Epidemiology and Biostatistics, College of Public Health, Zhengzhou University, Zhengzhou, Henan, China.

**Keywords:** carbapenemase genes, *Klebsiella pneumoniae*, transmission

## Abstract

The emergence of carbapenem-resistant *Enterobacteriaceae* made the treatment difficult, which has become a significant issue of public health. A sharp increase of carbapenem-resistance rate in *Klebsiella pneumoniae* was observed in a maternity and child health care hospital in Zunyi, China, in 2014.

In 2015 to 2016, carbapenem-resistant *Klebsiella pneumoniae* (CRKp) isolated from all the clinical samples were analyzed to identify the carbapenem-resistance genes. They were then fingerprinted in order to determine their genetic relationship. Clinical data such as usage of imipenem in 2012 to 2016 and the nosocomial infection surveillance data were analyzed.

Thirty-five isolates of CRKp out of 4328 various pathogens were obtained, and *bla*_NDM-1_ was identified to be the most common resistant gene present in the CRKp isolates. The fingerprint analysis identified 15 major clusters of CRKp isolates. The bacteria with close proximity relationship tended to be from the same wards. However, a few CRKp isolates from different wards were found to be genetically highly related. The clinical data showed a significantly higher usage of carbapenems in 2012 to 2013 before the CRKp rate sharply increased in 2014. The nosocomial infection surveillance showed an unexpectedly high rate of failures to meet the requirement of the hospital environment hygiene and hand hygiene in the neonatal ward.

The increasing isolation rate of CRKp was associated with poorly regulated usage of carbapenems, impropriate medical practices, and the poor hospital environmental hygiene and hand hygiene.

## 1 Introduction

*Klebsiella pneumoniae* (*K. pneumoniae*) is commonly known as an opportunistic bacterial pathogen in humans is responsible for a number of diseases such as respiratory tract infections, urinary tract infections, and bloodstream infections.^[1]^*K. pneumoniae* is also the most common cause of bloodstream infection resulting from hospital-acquired infections.^[2]^ According to the US statistics, the total annual cost for the hospital-acquired infections was 8.3 to 11.5 billion dollars in 2013 alone.^[3]^ In China, gram-negative bacteria are the third most common cause of hospital-acquired pneumonia,^[4]^ hence being a major potential risk of increases in the associated morbidity for hospitalized patients and health care cost. Furthermore, if the pathogens become resistant to commonly used antibiotics, they can increase mortality, especially in the case of carbapenem-resistant *Enterobacteriaceae* (CRE).^[5,6]^ Since an audit carried out in hospital revealed that there was a sharp increase of carbapenem resistance rates in *K. pneumoniae* in 2014, this study was initiated to analyze the clinical data of inpatients from 2012 to 2016, in order to understand the overall usage of carbapenems during the period in the hospital. The carbapenem-resistant *Klebsiella pneumoniae* (CRKp) isolates obtained from the clinical sample of 2015 to 2016 were analyzed to determine the genes responsible for carbapenem resistance as well as fingerprinting analysis to establish the genetic relationship among these isolates.

## 2 Materials and methods

### 2.1 Design

Prevalence of CRKp was investigated in clinical samples to identify a significantly increased rate. The clinical use of imipenem and the environmental factors recorded in the previous nosocomial infection surveillance several years before and during the period of the high prevalence of CRKp were analyzed to identify the possible cause of increase of CRKp rate. Deoxyribonucleic acid (DNA) fingerprint analysis of CRKp isolates from various wards was performed to establish the genetic relationship among these isolates.

### 2.2 Setting

Bacteria were isolated from clinical samples in the Maternity and Child Health Care Hospital, Zunyi, China in 2015 to 2016. Clinical data of inpatients who attended the hospital in 2012 to 2016 were retrieved and collected for statistical analysis.

### 2.3 Clinical specimens

All the non-duplicate CRKp isolates were isolated from the clinical samples collected from various wards by growing on Columbia Blood Agar during 2015 to 2016. The resistance to antibiotics imipenem was detected by Vitek 2 Compact (BioMérieux, France), then confirmed by imipenem E-test. These CRKp, with their imipenem minimum inhibitory concentrations being ≥4 μg mL^−1^, and the Carba NP test being phenotypical screening positive, were stored in 10 g/100 mL skim milk at −70°C before analysis.

### 2.4 Detection of carbapenemases genes and sequencing

The CRKp isolates stored at −70°C, were inoculated in Luria-Bertani medium and incubated at 37°C overnight and their genomic DNA was purified using Ezup Column Bacteria Genomic DNA Purification Kit (Sangon Biotech Shanghai. Inc. Shanghai, China). Carbapenemase genes (*bla*_SME_, *bla*_IMP_, *bla*_OXA-48_, *bla*_KPC_, *bla*_NDM_, *bla*_VIM_) were detected by using real-time polymerase chain reaction (PCR) with specific primers as shown in Table 1. The PCR conditions were pre-denaturing at 94°C for the 30 seconds followed by 40 cycles of 94°C 5 seconds for denaturing, 58 to 62°C 34 seconds for primer annealing and 72°C 30 seconds for product extension. At the end of amplification, the PCR products were melted at 95°C 15 seconds, 60°C 1 minute, 95°C 30 seconds, 60°C 15 seconds to determine the melt curve. Ten PCR products were randomly sequenced to confirm the identity of the PCR products.

**Table 1:** Primer sequences for carbapenemases genes.

Carbapenemases genes	Primer sequence
*bla*_SME_	F: 5’-TTAACACTGCAATCCCAGGAGA-3’R: 5’-CTACAACCCAATCAGCAGGAAC-3’
*bla*_IMP_	F: 5’-GCTTGATGAAGGCGTTTATGTT-3’R: 5’-CTGTCGCTATGAAAATGAGAGGA-3’
*bla*_OXA-48_	F: 5’-AGCAAAGGAATGGCAAGAAAAC-3’R: 5’-TAAAGGTAGATGCGGGTAAAAAT-3’
*bla*_KPC_	F: 5’-TTGATTGGCTAAAGGGAAAACACGAC-3’ R: 5’-GCCAGACGACGGCATAGTCATTT-3’
*bla*_NDM_	F: 5’-GCAGTCGCTTCCAACGGTTT-3’R: 5’-CAAGCTGGTTCGACAACGCA-3’
*bla*_VIM_	F: 5’-GTCGCATATCGCAACGCAGT-3’R: 5’-CGACGCGGTCGTCATGAAAG-3’

### 2.5 ERIC-PCR

Enterobacterial repetitive intergenic consensus (ERIC)-PCR was performed on the purified genomic DNA using ERIC primers (ERIC-1R 5′-ATGTAAGCTCCTGGGGATTCAC-3′, ERIC-2 5′-AAGTAAGTGACTGGGGTGAGCG-3′).^[7,8]^ The DNA was amplified as following: 94°C 1 minute, 52°C 1 minute, 68°C 8 minutes for 38 cycles; 65°C 16 minutes for 1 cycle. Then the PCR product was electrophoresed on 2 g/100 mL agarose gel containing 0.25 μg mL^−1^ of ethidium bromide. These gels were photographed in the Gel Doc XR+ system (Bio-Rad Laboratories. Inc., California, USA).

### 2.6 Clinical data collection

The clinical data were retrieved from the database recorded in the department of pathology and other relevant departments of the hospital for analysis. These included the inpatients from 2012 to 2016, and their detection of 4 imipenem-resistant *Enterobacteriaceae* pathogens, *Escherichia coli* (*E. coli*), *K. pneumoniae*, *Enterobacter cloacae*, and *Klebsiella oxytoca* (*K. oxytoca*). Data were analyzed to determine whether these patients having received imipenem treatment, and other β-lactam in addition to imipenem, had their infection confirmed by isolation of pathogens as well as whether those pathogens were extended-spectrum beta-lactamases (ESBLs)-producing, which is one of the criteria to justify the use of imipenem.

### 2.7 Nosocomial infection surveillance

Nosocomial infection surveillance data from 2012 to 2016 were retrieved from the hospital database, which includes the bacteria count of air and doctors’ and nurses’ hands from the wards in which CRKp cases were identified. The allowable range of bacterial count for air and hands were set to be ≤200 cfu/m^3^ and ≤5 cfu/cm^2^, respectively, according to the guidelines in China, which are outlined in the WS/T 313-2009 for the hand hygiene for healthcare workers and in the GB15982-1995 for disinfection in hospitals. Briefly, the subject's finger tips were sampled by rubbing with a cotton swab soaked in sterile eluent containing neutralizing agent. The swap was then put into a 10 mL sterile eluent tube containing the corresponding neutralizer. The sample tube was oscillated on the mixer for 20 seconds and duplicate samples were inoculated on agar media plates, which were then incubated at 37°C for 48 hours allowing the formed bacterial colonies to be counted.

### 2.8 Genetical analysis of CRKp isolates

The fingerprint bands of CRKp produced by ERIC-PCR were analyzed and the cluster data table and the relative distance and the dendrogram were generated by using IBM SPSS Statistics software. The differences of genotypes are displayed according to the relative distance among strains.

### 2.9 Statistical analysis

The trend of quarterly changes and the number of 4 kinds of imipenem-resistant *Enterobacteriaceae* was analyzed by Chi-square test for trend, and the correlation between the overall proportion of imipenem use and the number of the 4 CRE was analyzed by Spearman rank correlation by using GraphPad Prism 8.0.1 (California, USA). A bilateral *P* value of less than .05 is considered statistically significant.

### 2.10 Ethics declarations

The study is approved by the Medical Ethics Committee of Zunyi Maternity and Child Health Care Hospital (Ref No. EC-2014S-001). Informed consent for the collection of clinical samples was obtained from either parent or legal guardian of the subjects. Clinical data retrieved from the database recorded in the department of pathology and other relevant departments of the hospital have been waived the need for informed consent for the clinical data used in this study by Medical Ethics Committee of Zunyi Maternity and Child Health Care Hospital.

## 3 Results

### 3.1 *bla*_NDM-1_ is the most common carbapenemase gene in the CRKp isolates

Totally 35 CRKp isolates out of 4328 various pathogens were analyzed from the neonatal ward, pediatric wards 2 and 3 as well as pediatric intensive care unit (PICU), representing an overall ∼0.81% detection rate. Most of the CRKp isolates (25/35, ∼71%) were from the neonatal ward with 6 from the PICU (∼17%) and 4 (∼11%) from the 2 pediatric wards. Almost all the CRKp strains were isolated from the sputum samples, except for 2 with 1 from tracheal intubation (ID# 9 from the neonatal ward) and the other from the blood (ID# 35 from PICU), as enlisted in Table 2.

**Table 2:** Detection of carbapenemase genes in carbapenem-resistant *Klebsiella pneumoniae* isolates from specified wards^∗^.

			Carbapenemase gene					
ID^#^	Inpatient ward	Source of bacterial isolates	*bla*_KPC_	*bla*_NDM-1_	*bla*_OXA-48_	*bla*_IMP_	*bla*_VIM_	*bla*_SME_
73	Pediatric ward-3	Sputum	–	+	–	–	+	–
11	Pediatric ward-2	Sputum	–	+	–	–	–	–
70	Pediatric ward-2	Sputum	–	+	–	–	–	–
71	Pediatric ward-2	Sputum	–	+	–	–	–	–
35	PICU	Blood	–	–	–	–	–	–
36	PICU	Sputum	–	+	–	–	–	–
65	PICU	Sputum	–	+	–	+	–	–
75	PICU	Sputum	–	+	–	–	–	–
76	PICU	Sputum	–	+	–	–	–	–
77	PICU	Sputum	–	+	–	–	–	–
9	Neonatal ward	Tracheal intubation	–	+	–	–	–	–
10	Neonatal ward	Sputum	–	+	–	–	–	–
12	Neonatal ward	Sputum	–	+	–	–	–	–
13	Neonatal ward	Sputum	–	+	–	–	–	–
19	Neonatal ward	Sputum	–	+	–	–	–	–
20	Neonatal ward	Sputum	+	+	–	–	–	–
21	Neonatal ward	Sputum	+	+	–	–	–	–
22	Neonatal ward	Sputum	–	+	–	–	–	–
23	Neonatal ward	Sputum	–	+	–	–	–	–
24	Neonatal ward	Sputum	–	+	–	–	–	–
34	Neonatal ward	Sputum	–	+	–	–	–	–
38	Neonatal ward	Sputum	–	+	–	–	–	–
45	Neonatal ward	Sputum	–	–	–	–	–	–
46	Neonatal ward	Sputum	–	–	–	–	–	+
49	Neonatal ward	Sputum	–	+	–	–	–	–
50	Neonatal ward	Sputum	+	+	–	–	–	–
51	Neonatal ward	Sputum	–	+	–	–	–	–
53	Neonatal ward	Sputum	–	+	–	–	–	–
54	Neonatal ward	Sputum	–	+	–	–	–	–
59	Neonatal ward	Sputum	–	+	–	–	–	–
60	Neonatal ward	Sputum	–	+	–	–	–	–
61	Neonatal ward	Sputum	–	+	–	–	–	–
62	Neonatal ward	Sputum	–	+	–	–	–	–
63	Neonatal ward	Sputum	–	+	–	–	–	–
72	Neonatal ward	Sputum	–	+	–	–	+	–

Twenty-six out of 35 CRKp isolates were detected to contain *bla*_NDM-1_ gene (Table 2). Moreover, 10 randomly chosen *bla*_NDM-1_ PCR products were sequenced, and the results confirmed that they were all correct specific products of the *bla*_NDM-1_ gene.

### 3.2 CRKp with closely related genotypes can be detected in patients from different wards

**Figure 1:**
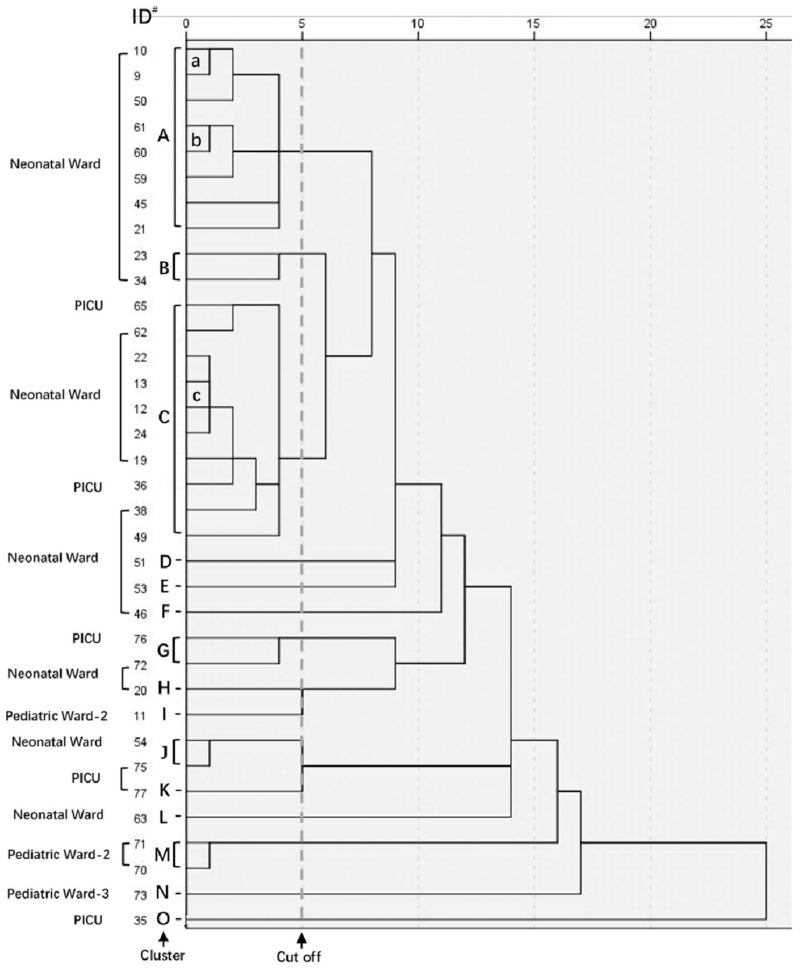
Dendrogram of 35 imipenem-resistant *Klebaiella pneumoniae*. Dendrogram using average (between-group) linkage shows the genetic relationship among the isolates based on the ERIC-PCR results. The number 0 to 25 at the top horizontal scale is the relative distance between the strains. The numbers of vertical axis at the left of figure are sample numbers and the capital letters A–O indicate the clusters of isolates which have a high genetic similarity (<5 relative distance). The lower case letters a–c indicate the sub-clusters of the isolates within a cluster, which have the lowest relative distance. ERIC = enterobacterial repetitive intergenic consensus, PCR = polymerase chain reaction, PICU = pediatric intensive care unit.

ERIC-PCR analysis divided the 35 CRKp isolates into 15 major clusters (A–O) (Fig. 1). In each cluster, the isolates had high genotypic similarity with a cutoff of <5 by ERIC-PCR. Ten clusters were isolated from the neonatal ward (A, B, C, D, E, F, G, H, J, L), 5 from PICU (C, G, J, K, O), 2 from pediatric ward-2 (I, M), 1 from pediatric ward-3 (N). More than half of CRKp isolates belonged to clusters A (8/35) and C (10/35). Besides, ERIC-PCR results revealed 12 strains in 5 clusters or sub-clusters with the highest genetic similarity, being sub-clusters a (10, 9), b (61, 60), c (22, 13, 12 and 24) from neonatal ward and cluster M (71, 70) from PICU, as well as cluster J (54,75) with 1 (54) from neonatal ward and the other (75) from PICU. It was noted that most of the genetically related isolated were from the same wards, but there were a few clusters of isolates found in different wards, such as the clusters C, G, and J being found in PICU and neonatal wards.

### 3.3 Imipenem overuse prior to the increase of CRKp prevalence

**Figure 2:**
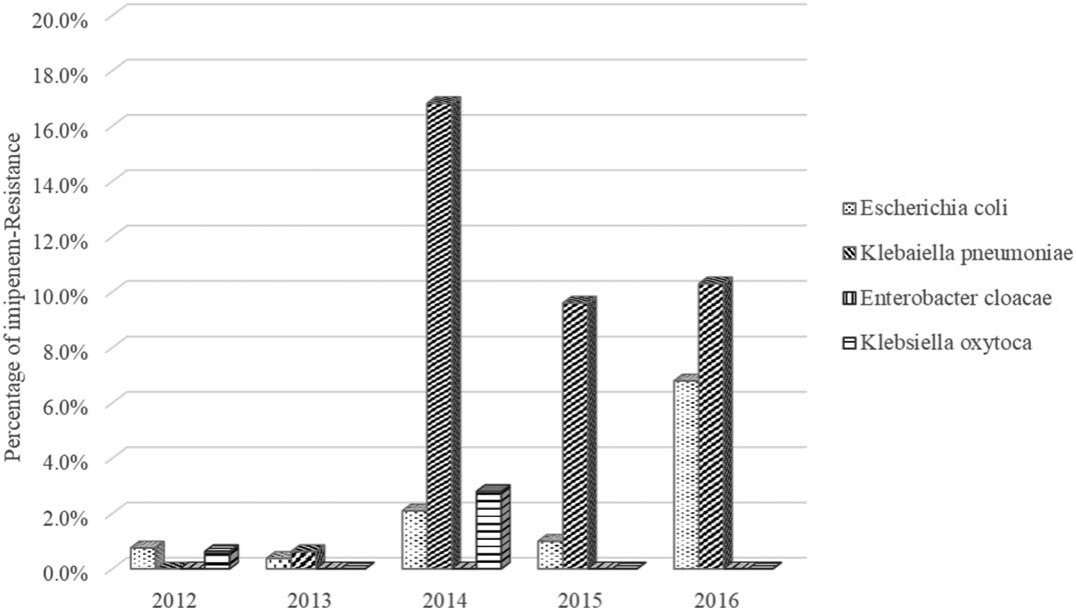
The percentage of imipenem-resistant Enterobacteriaceae pathogens. Rates (%) of imipenem resistant isolates of 4 commonly known bacterial pathogens (*Escherichia coli, Klebaiella pneumoniae, Enterobacter cloacae*, and *Klebsiella oxytoca)* are shown.

As shown in Figure 2, the imipenem-resistant *K. pneumoniae* were barely detected in 2012 and 2013, but >16% isolated *K. pneumoniae* were found to be imipenem resistant in 2014, and the imipenem resistance rate in *K. pneumoniae* isolates remained high, at ∼10%, in the following 2 years. The imipenem resistant *E. coli* were also found to be increased in 2014 and 2015 (<2%) and further increased to >6% in 2016, while no apparent imipenem resistance was found in the *Enterobacter cloacae* and *K. oxytoca* isolated in 2012 to 2016. Statistical analysis of the original data (chi-square test) showed that the trend of the imipenem-resistance rate in *K. pneumorniae* and *E. coli* were significantly different followed by the change of time (quarter) (χ2 = 23.98, *P* < .001 and χ2 = 6.46, *P* = .01 respectively), whereas there was no statistically significant difference in imipenem-resistance in *E. cloacae* and *K. oxytoca.*

Analysis of the clinical data recorded in 2012 to 2016 (Table 3) shows that in 2012 and 2013, there were 111 and 137 inpatients, respectively, who had been treated with imipenem. Among them, 87 and 85 were either bacterial pathogen negative or not tested for bacterial pathogens at all, representing 78% and 62%, respectively, of the patients having received the imipenem treatment. Among the pathogen positive and imipenem-treated inpatients, approximately 27% of them in 2012 and 2013, had their pathogens determined to be ESBLs producing, which should be considered to be qualified for the use of imipenem. In the following years, the overall number of imipenem-treated inpatients decreased to 92, 54, and 48 in the years 2014, 2015, and 2016, respectively. Among them, although the percentage of the pathogen-positive inpatients increased to 65%, 53%, and 58%, respectively, the percentage of those with their pathogens determined to be ESBLs producing remained similar, being 31%, 27%, and 32%, respectively (Table 3). According to Spearman rank correlation analysis, the results showed that there was a linear negative correlation between the use of imipenem and the percentage of imipenem-resistance in *K. pneumoniae* (r = –0.5593, *P* = .02), and there was no correlation between the use of imipenem and the percentage of imipenem-resistance in *E. coli*, *E. cloacae*, and *K. oxytoca* (*P* > .05). The trend of positive rate of pathogen detection in patients treated with imipenem per year was significantly different (χ2 = 24.49, *P* < .001). Interestingly, there were linear correlations between time (year) and the total number of imipenem treated patients and the number of imipenem treated patients with pathogens detected negative or test not done (*P* = .04 and *P* = .02, respectively). Both the number of imipenem treated patients and imipenem treated patients with pathogens detected negative or test not down decreased gradually with year.

**Table 3:** Imipenem resistant rate and imipenem usage (n/%).

			Imipenem treated patients^†^	
Year	Discharged patients (n)	Imipenem treated patients^∗^	Pathogen negative/ND	Pathogen positive
2012	11634	111 (0.76%)	87 (78%)	24 (22%)ESBLs^+^: 8 (27%)^‡^
2013	14311	137 (0.97%)	85 (62%)	52 (38%)ESBLs^+^: 14 (27%)
2014	15287	92 (0.60%)	32 (35%)	60 (65%)ESBLs^+^: 19 (31%)
2015	17396	54 (0.32%)	25 (47%)	29 (53%)ESBLs^+^: 8 (27%)
2016	23060	48 (0.20%)	20 (42%)	28 (58%)ESBLs^+^: 9 (32%)

### 3.4 Neonatal ward was beyond the allowable range of nosocomial infection

Table 4 showed that the monthly bacteria count in the air of the neonatal ward were beyond the allowable range 3 times in 2012 and increased to 9, 10, 7, and 7 times in years 2013 to 2016, respectively. There was 2 times each year of failure to meet the standard of bacterial count on hands in the neonatal ward in 2014 and 2016. The bacterial count in the air of PICU ward also failed to meet the standard 3 times each year in 2013, 2014, and 2016, respectively and once in 2015. The pediatric wards were found to be overall compliant with the hygiene standards in the air and on hand with occasional failures for hand hygiene.

**Table 4:** Failure to meet the hygiene standards: monthly results of nosocomial infection surveillance^∗^.

Wards	2012	2013	2014	2015	2016
Neonatal ward (air)	3	9	10	7	7
Neonatal ward (hands)	0	0	2	0	2
Ped-1 (air)	0	0	0	0	0
Ped-1 (hands)	0	1	1	0	0
Ped-2 (air)	0	0	0	0	0
Ped-2 (hands)	0	0	1	1	0
PICU (air)	0	3	3	1	3
PICU (hands)	0	0	2	0	2
Ped-3 (air)^†^	–	–	–	0	0
Ped-3 (hands)^†^	–	–	–	2	0

## 4 Discussion

The growth of the imipenem resistant rate in *K. pneumoniae* is the fastest among the all species of the Enterobacteriaceae in the hospital in recent years, followed by *E. coli* (Fig. 2). The carbapenem resistance can be caused by the acquisition of plasmids with various carbapenemase genes. Six carbapenemase candidate genes were analyzed in the CRKp isolates in this study. The most frequently identified gene is *bla*_NDM-1_, which is different from the previous findings in China that the common epidemic genotype is found to be *bla*_KPC_.^[9,10]^

In 2 CRKp isolates (ID^#^ 35 and ID^#^ 45) (Table 2), none of these 6 carbapenemase genes was detected, probably because their carbapenem resistance was conferred by other than these 6 resistance genes, or by other mechanisms of resistance, such as porin loss and/or efflux pump activation.^[11]^ Carbapenemases belong to β-lactamases with the capacity to hydrolyze or inactivate carbapenems. As per the Ambler molecular classification, the carbapenemases are divided into class A, B, and D.^[12]^ Serratia marcescens enzyme and *K. pneumoniae* carbapenemase belong to class A carbapenemases. Verona imipenemase, imipenemase, and New Delhi metallo-β-lactamase belong to class B carbapenemases. Class D carbapenemases have been described among 4 sub-families of oxacilinase type β-lactamases.^[13,14]^ Because of the different resistant mechanism, doctors can choose the appropriate treatment according to the prevalence of the specific carbapenemase gene.^[15]^ The behavior of the bacterial pathogens can change as a result of genetic mutations by insertion/deletion of DNA fragment, homologous recombination, spontaneous induction of the SOS response, and replication-transcription conflict to adapt to the environment and to resist the attack of antibiotics,^[16,17]^ consequently leading to the development of antibiotic resistance including resistance to carbapenem. This is a slow process, and it is estimated that spontaneous mutations occur at a rate of 10^−10^ to 10^−9^ per nucleotide per generation for most of the bacteria under certain growth conditions.^[16]^ In this study, the resistance rates of CRKp jumped swiftly from 0.6% in 2013 to 16.9% in 2014 (Fig. 2), which was a unusual event and warranted an urgent study to identify the mechanism of this growth in order to develop an effective strategy to prevent further spreading, ultimately reducing the presence of CRKp in the affected hospital.

ESBLs are enzymes produced by some bacterial pathogens that are able to hydrolyze 3^rd^ generation antibiotics such as cephalosporins and aztreonam, leading to nasty resistance to commonly used antibiotics. Carbapenems are beta-lactam antibiotics that have a broad spectrum of activities against many gram-positive and gram-negative, aerobic and anaerobic organisms. Imipenem is the first antibiotic of carbapenems approved as a potent broad-spectrum antibiotic for treatment in 1985. Subsequently, the other carbapenems such as meropenem, ertapenem were developed.^[18]^ They are highly stable to resist the hydrolysis by the ESBLs and AmpC beta-lactamases, which are considered to be the “gold standard” treatment for serious ESBL producing pathogen infections^[19]^ and infection of complex bacteria with multidrug resistance. Unfortunately, they were tended to be prescribed for conditions suspected to be an infection but without pathogens identified or for cases which were not confirmed whether an infection was involved. According to the analysis of the clinical data in this study (Table 3), 0.76% and 0.97% of all the discharged patients were treated with imipenem in 2012 and 2013, among whom, all of them were pediatric patients, most of them had no pathogen detected, or no test performed to detect pathogens. Even for the pathogens isolated from a small percentage of patients who received imipenem treatment, only approximately one-third of the pathogens identified were confirmed to be ESBLs-producing, and most of them were non-ESBLs-producing bacteria or non-enterobacteriaceae bacteria, such as *Haemophilus influenzae*, *Streptococcus pneumoniae,* etc (Table S1, Supplemental Digital Content), which should have been treated by antibiotics other than carbapenem.

During this study, an irregular combination of medications was also found, including cases who received imipenem combined with other β-lactams. It appeared that prescription of carbapenem antibiotics was not well restricted, and there was a lack of robust justification for using carbapenem via collective discussion with experts during those years. Fortunately, the relevant guidelines are now in place. However, the overuse of antibiotics not only occurred at this hospital but also in other hospitals in China and other countries as having been previously reported.^[20–22]^ The average annual growth rate of defined daily doses of carbapenem antibiotics of 151-grade general tertiary hospitals in China from 2011 to 2014 was 17.67%.^[20]^ At the same time, the rates of imipenem resistance in *K. pneumoniae* trended to increase from 8% to 10.5% in China.^[21]^ In Thailand, the quarterly CRE incidence increased significantly from 3.37 per 100,000 patient-days in the last quarter of 2011 to 32.49 per 100,000 patient-days in the last quarter of 2016. The quarterly hospital-wide carbapenem consumption increased by 1.58 defined daily doses per 1000 patient-days in the corresponding time.^[22]^ Obviously, heavy use of this antibiotic is considered to favor selecting resistance mutation and lead to the emergence of CRKp. Schroeder et al^[16]^ has detailed the mechanism of antibiotic resistance previously. A meta-analysis has revealed that antibiotic usage can lead to the resistance mutation, even in animal husbandry where there is a positive association between bacterial resistance and antibiotic consumption in farm animals.^[23–26]^ Interestingly, in this study, our statistical analysis showed that the irregular use of imipenem in 2012 to 2013 (Table 3) had a delayed, rather than immediate effect in causing the sudden increase of CRKp which occurred in 2014 and then gradually decreased (Fig. 2) along with the irregular use of imipenem being reduced after 2014.

Furthermore, our data analysis has shown that, firstly, most of the inpatients in the neonatal wards had no corresponding clinical symptoms, albeit they had the highest rate of CRKp pathogen isolation. This suggests that the CRKp in the neonatal patients are likely colonizing in the body, but not responsible for the infection. Secondly, most of the bacteria were isolated from the second time sputum detection from the same inpatient, with the first sputum examination being negative for pathogens. This is highly likely that they acquired the bacteria in the hospital, and these bacteria probably were spreading in the same ward, even between the different wards. The speculation has been supported by the cluster analysis of the pathogen genetic fingerprinting results. Cluster analysis showed that most of the highly similar genotypes bacteria were isolated from the same wards, especially sub-clusters a, b, c, and clusters J and M which were most closely related, suggesting that they were likely spreading between the different inpatients at the same ward. Moreover, the highly related pathogens were also isolated from different wards, such as clusters C, G, and J, they were isolated from neonatal ward and PICU wards. The highly close genetic relationship among these pathogens suggests that they were spreading between the different wards as a result of the reproduction of the same bacterial strain. This speculation is supported by the observation that most of these pathogens carry the same resistant gene *bla*_NDM-1_.

It is known that bacteria can acquire external genetic material through transformation, transduction, and conjugation. Conjugation is, in particular, a very efficient approach of inter-bacterial gene transfer that involves cell-to-cell contact,^[27]^ found to be a key mechanism for the transfer of antibiotic-resistant genes between different bacteria. Hospital is prone to contamination of various pathogens, where strict guidelines or rules must be observed for preventing nosocomial infections. A meta-analysis examining 13 risk factors for CRE acquisition has shown that the use of medical devices and carbapenem use in hospitals have the highest association with the CRE acquisition. There are a number of other contributing factors to be noted, such as other antibiotic exposure, underlying disease or condition, invasive procedures, medical devices, intensive care unit admission, patient demographic characteristics, exposure to hospital care, mechanical ventilation.^[28]^ Indeed, our analysis of the nosocomial infection surveillance data from 2012 to 2016 in this study showed that the bacteria count in the air of neonatal ward often exceeded the allowable range every year, for example, it exceeded the limit in 10 of 12 surveys in 2014, indicating that bacterial count in the air of the neonatal ward was mostly out of control, at least, during the continuous period of time in 2014 and potentially for a long time before and after the year. The pathogens, such as CRKp, could be inhaled into an inpatients’ respiratory tract, or it could settle onto the surfaces of body or objects and then acquired by patients during the hospitalization. We have also examined the bacterial count on the hands of doctors or nurses. The data (Table 4) showed that there were several times out of 12 monthly surveys that the on-hand bacterial count excessed in 2012 and 2016. These data could be underestimated considering that the subjects to be surveyed were notified in advance and prepared for the test, who would have paid more than usual attention to the hand hygiene until detection. Observations and personal conversations indicated that the medical staff tended to not do hand disinfection nor changed gloves between examinations of patients. It is highly likely that the hands contaminated with pathogens would transmit them to everywhere they touched, including the patients, other medical staff, medical devices, even objects around the hospital. The transmission can likely further increase the chance for the antibiotic-resistant genes to be transferred among the bacteria existing in the hospital environment. The limitation of this study is that the other environmental substances in and around the hospital were not thoroughly examined for the existence of bacteria with antibiotic resistance genes, such as soil, water, and waste, which could be important contributing factors.^[29]^

In conclusion, this study illustrates the molecular characterization and prevalence of CRKp in the maternity and child health care hospital in Zunyi, China. Base on the analysis, the swift increase in the CRKp was a result of irregular use of imipenem in the previous years and the poor hospital environmental hygiene and hand hygiene played a role in facilitating the spread of the *bla*_NDM-1_ gene containing pathogen bacteria among the pediatric wards. The conclusive information should be useful in assisting optimizing the guidelines for controlling the bacterial infection in hospitals as well as helpful for the doctors to make a decision in treating patients with infections.

## Author contributions

**Conceptualization:** Yuxin Yang.

**Data curation:** Yuxin Yang, Jia Liu, Jing Lu.

**Formal analysis:** Yuxin Yang.

**Funding acquisition:** Yuxin Yang.

**Validation:** Zongsu Min.

**Writing – original draft:** Yuxin Yang, Murad Muhammad, Hanting Liu, Lei Zhang.

**Writing – review & editing:** Zhonglin Chai.

## Supplementary Material

Supplemental Digital Content
