# Supplementary material for: Factors behind the prevalence of carbapenem-resistant Klebsiella pneumoniae in pediatric wards
Source: Medicine (Baltimore). 2021 Sep 10;100(36):e27186. doi: 10.1097/MD.0000000000027186 (PMC8428699; doi:10.1097/MD.0000000000027186)
Supplement: Supplemental Digital Content [file medi-100-e27186-s001.docx]

**Factors behind the prevalence of carbapenem-resistant *Klebsiella Pneumoniae* in Pediatric Wards**

Yuxin Yang MM^1,5*^, Jia Liu MM^2^, Murad Muhammad PhD^3^, Hanting Liu PhD^6^, Zongsu Min MBBS^1^, Jing Lu MD^4^, Lei Zhang PhD^6,7,8,9^, Zhonglin Chai PhD^5*^

^1^ *Department of Pathology,* *Zunyi* *Maternity and Child Health Care Hospital, Zunyi, Guizhou, China*

^2^*Department of Pharmacy, Zunyi Maternity and Child Health Care Hospital, Zunyi, Guizhou, China*

^3^*Department of Surgery (RMH), University of Melbourne, Melbourne, Victoria, Australia*

^4^*Department of Breast Surgery, Guizhou Provincial People's Hospital, Guiyang, Guizhou, China*

^5^*Department of Diabetes, Central Clinical School, Monash University, Melbourne, Victoria, Australia*

*^6^China-Australia Joint Research Center for Infectious Diseases, School of Public Health, Xi’an Jiaotong University Health Science Center, Xi’an, Shanxi, China.*

*^7^Artificial Intelligence and Modelling in Epidemiology Program, Melbourne Sexual Health Centre, Alfred Health, Melbourne, Australia*

*^8^Central Clinical School, Faculty of Medicine, Monash University, Melbourne, Australia*

*^9^Department of Epidemiology and Biostatistics, College of Public Health, Zhengzhou University, Zhengzhou, Henan, China.*

*Corresponding author.

Zhonglin Chai, E-mail address: [zhonglin.chai@monash.edu](mailto:zhonglin.chai@monash.edu)

Yuxin Yang, E-mail address: [yangxx_123@126.com](mailto:yangxx_123@126.com)

**Supplementary table 1: Bacterial pathogen species isolated from the imipenem-treated inpatients**

| Name of pathogens | 2012 | 2013 | 2014 | 2015 | 2016 |
| --- | --- | --- | --- | --- | --- |
| *Acinetobacter calcoaceticus* | - | 1 | - | - | - |
| *Burkholderia cepacia* | - | 1 | - | - | - |
| *Candida* | - | 2 | 1 | 1 | - |
| *Citrobacter freundii* | 1 | - | - | - | - |
| *Enterobacter aerogenes* | - | 1 | - | - | - |
| *Enterobacter cloacae* | 1 | 2 | 1 | - | - |
| *Escherichia coli* | 7 | 9 | 9 | 2 | 6 |
| *Haemophilus ducreyi* | - | 1 | - | - | - |
| *Haemophilus influenzae* | 1 | 2 | 8 | 1 | 2 |
| *Haemophilus parainfluenzae* | 1 | 4 | - | - | - |
| *Klebsiella oxytoca* | 1 | - | 3 | 1 | - |
| *Klebsiella pneumoniae* | - | 5 | 10 | 5 | 4 |
| *Klebsiella terrigena* | 1 | - | - | - | - |
| *Moraxella catarrhalis* | - | 2 | 6 | 5 | 2 |
| *Pantoea agglomerans* | - | 1 | - | - | - |
| *Pseudomonas aeruginosa* | - | 1 | - | - | - |
| *pseudomonas fluorescens* | - | 3 | - | - | - |
| *Pseudomonas putida* | - | 1 | - | - | - |
| *Salmonella typhi* | 1 | - | - | - | - |
| *Serratia fonticola* | 6 | 1 | - | - | - |
| *Serratia liquefaciens* | - | 1 | 2 | - | - |
| *Serratia marcescens* | 1 | - | 1 | 1 | - |
| *Serratia odorifera* | 1 | - | - | - | - |
| *Staphylococcus aureus* | 1 | - | 1 | 8 | 3 |
| *Staphylococcus epidermidis* | - | 1 | - | 2 | 1 |
| *Staphylococcus haemolyticus* | 1 | 4 | 3 | 1 | 1 |
| *Staphylococcus lugdunensis* | - | 3 | 4 | - | - |
| *Staphylococcus hominis* | - | 4 | 5 | 1 | 4 |
| *Enterococcus faecium* | - | - | - | - | 2 |
| *Enterococcus faecalis* | - | - | - | - | 1 |
| *Streptococcus pneumoniae* | - | 2 | 7 | 1 | 2 |
| Total | 24 | 52 | 60 | 29 | 28 |

-: No corresponding pathogens were detected.

Legends:

Supplementary Table 1: This table shows the bacterial pathogen species and how many times each of these species were detected from the imipenem treated patients each year in 2012-2016.
